# Supplementary material for: Transcriptome and chromatin landscape of iNKT cells are shaped by subset differentiation and antigen exposure
Source: Nat Commun. 2021 Mar 4;12:1446. doi: 10.1038/s41467-021-21574-w (PMC7933435; doi:10.1038/s41467-021-21574-w)
Supplement: Supplementary file 1 — Supplementary Information [file 41467_2021_21574_MOESM1_ESM.pdf]

Supplementary Figure 1

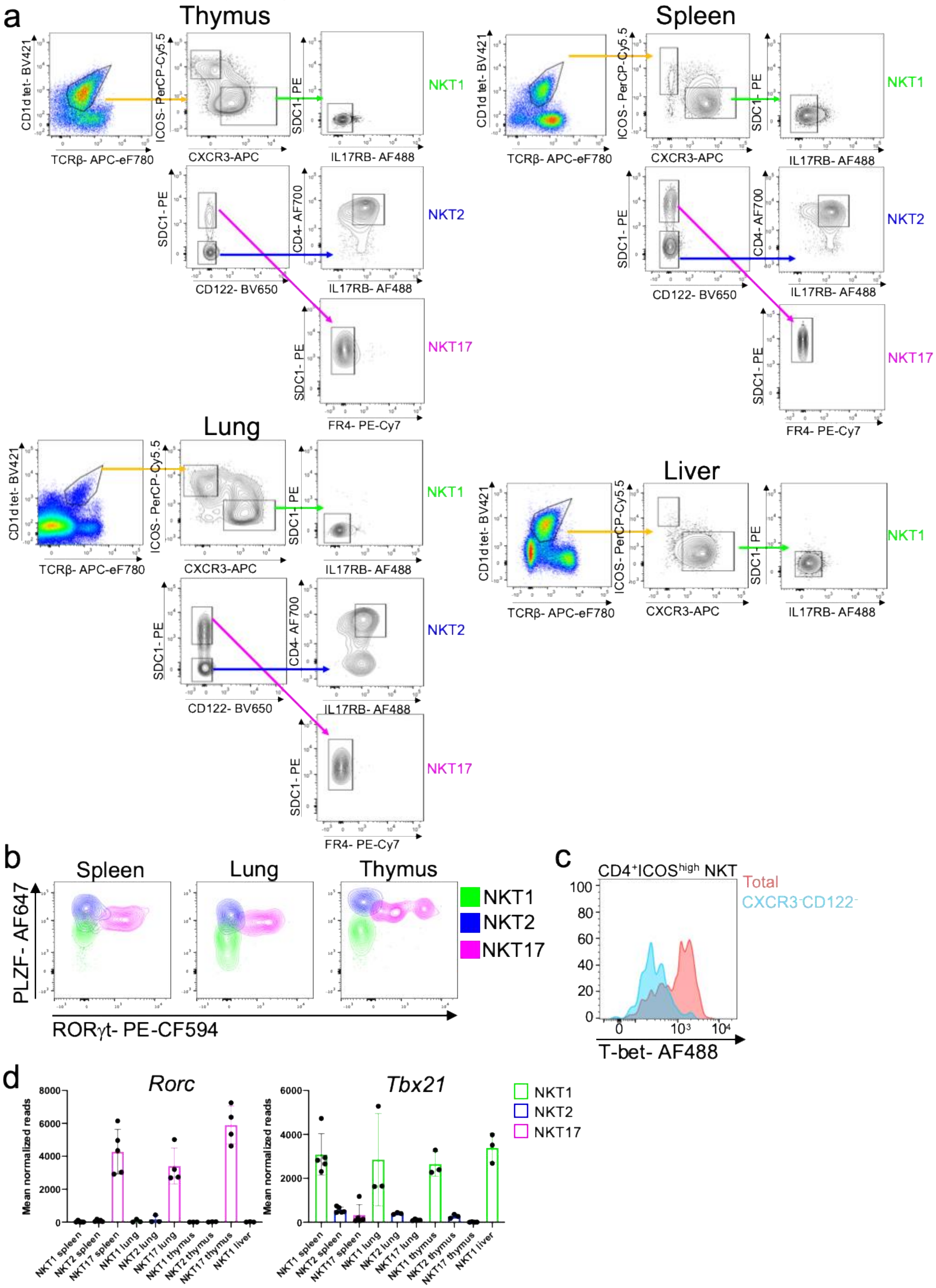

Supplementary Fig. 1. iNKT cell subset gating. a. Example of gating for isolating iNKT cell subsets from lymphocyte- and live-gated, cell suspensions enriched for *i*NKT cells as described in Methods. Note that some $ICOS^{high}$ cells express relatively high levels of CXCR3. Representative examples shown are from the thymus, spleen, lung and liver; one of five independent sorts. b. Expression of transcription factors PLZF and RORγt as measured by representative flow cytometry in spleen, lung, and thymus CD1d-αgalcer-tetramer-binding, $TCR\beta^+$ cells selected to be NKT1($CXCR3^+$, $ICOS^{low}$, $SDC1^-$), NKT2 ($CXCR3^-$, $ICOS^{high}$, $CD4^+$, $SDC1^-$), and NKT17 ($CXCR3^-$, $ICOS^{high}$, $CD4^-$, $SDC1^+$). Results are representative of cell suspensions from two mice stained with this exact panel. The staining of all of the panel reagents was confirmed in samples from at least eight mice examined in at least four independent experiments. c. Histogram overlay of staining for T-bet in $CD4^+$, $ICOS^{high}$ iNKT splenocytes with removal (cyan) or without removal (pink) of cells staining with antibodies specific for CXCR3 or CD122, which were combined into one channel to facilitate simultaneous staining for both cell surface markers and transcription factors to define iNKT cell subsets and identify more uniformly a T-bet^{low} population. Data are representative of samples from fourteen mice examined in seven independent experiments. d. Normalized reads with means +/- SD for *Rorc* and *Tbx21* as determined by RNA-seq for cells sorted as described in Fig. Supp. 1a. n=5 for the spleen samples, n=4 for NKT17 lung and thymus, and n=3 for all other samples.

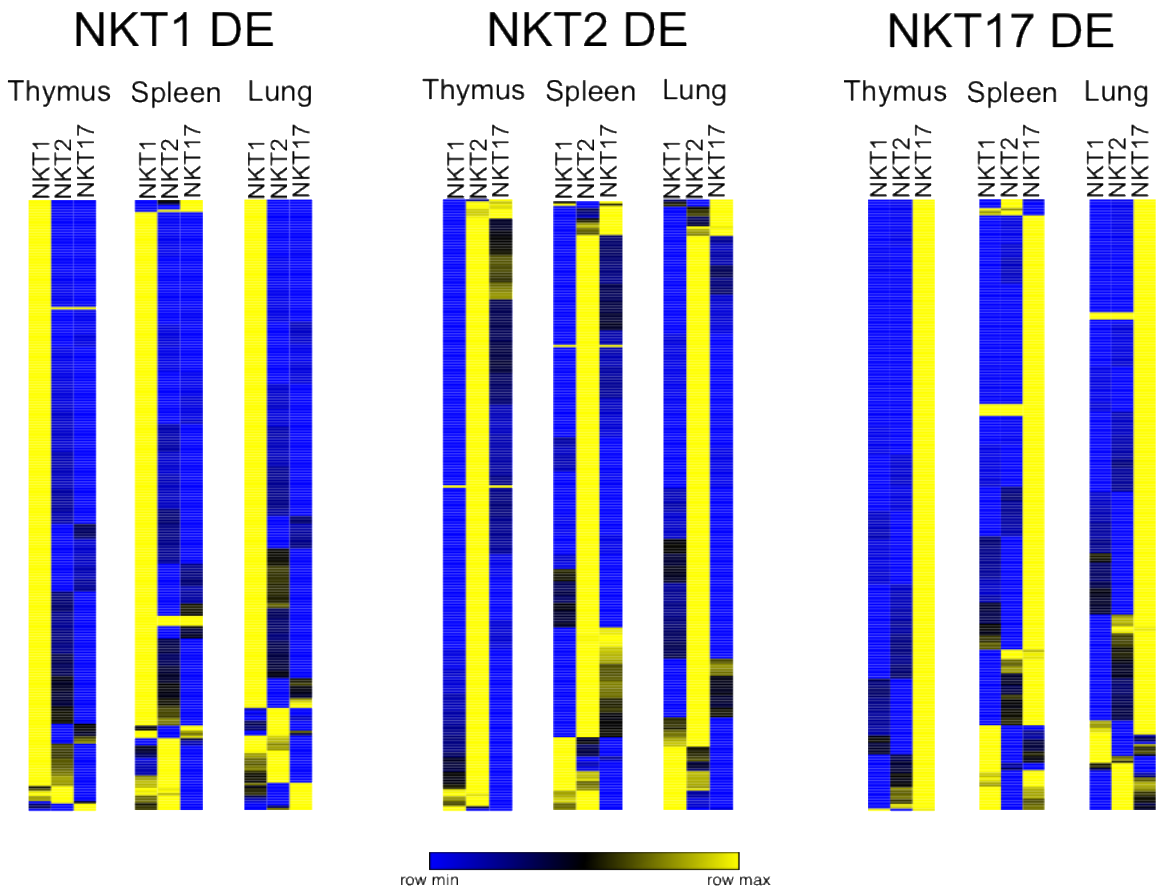

Supplementary Fig. 2. Reproducibility of thymic iNKT cell subset gene signatures. Thymic iNKT cell subset transcriptomic signatures, as previously defined[1], were compared to the data in this study. Differentially expressed (DE) genes from the earlier study, defined by comparing end subset to the other two, and having > 2-fold difference in mean reads and adjusted p = 0.05 (DESeq2 with Benjamini-Hochberg correction), from the earlier study were compared to the results from this study.

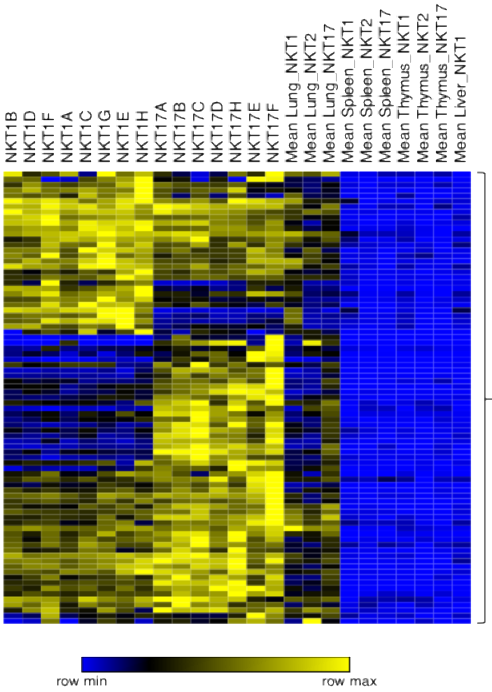

2810459M11Rik	Got1	Pim1
2900060B14Rik	Gpr116	Plaur
4930417O13Rik	Gpr132	Plcxd2
5430416N02Rik	Gprc5a	Plk2
Abcb1b	Gramd3	Pmp22
Adrb2	H3f3b	Polg2
Arid5a	Hdc	Prdm2
Arl5b	Hey1	Rab4a
Arl5c	Hilpda	Ramp3
Arrdc3	Hist1h1c	Rgs1
Atf3	Hist1h2b	Rnf125
Atf4	c	Rnf19a
Atp2b1	Hspa1a	Rrad
Bambi	Hspa1b	Samd11
Bcl2a1b	Ier5l	Samd8
Bcl2l11	Ifngr2	Samsn1
Btg2	Ifrd1	Scgb1a1
C920009B18Rik	Il1rl1	Serinc1
Cav1	Ipmk	Serpinb6b
Ccrl2	Irg1	Sgip1
Cd69	Isca1	Sgtb
Cdkn1a	Isy1	Sik1
Chrne	Kctd12	Snx18
Cldn5	Kdm6b	Spty2d1
Clk1	Klf4	Sult2b1
Cpne8	Klf6	Tgif1
Csrnp1	Lmn	Thbd
Ctla2a	Malt1	Tigit
Ctla4	Map1lc3a	Tinf2
Cwc25	Metrnl	Tiparp
Dennd4a	Muc1	Tmem64
Dgat1	Mxd1	Tnfrsf10b
Dnajb4	Naa38	Tnfrsf1b
Dnajb9	Neurl3	Tob1
Dusp5	Nfil3	Tob2
Emp1	Nfkbia	Trim43b
Ets2	Nfkbid	Trmt61b
Fasl	Nfkbiz	Trp53inp2
Fos	Nr4a1	Tsc22d3
Fosb	Nr4a2	Tspan7
Fosl2	Nr4a3	Tubb2a
G530011O06Rik	Obfc2a	Vgll4
Gabpb1	Odc1	Zbtb10
Gadd45b	P2ry10	Zc3h12a
Gm11974	Pard6a	Zc3hav1
Gm11978	Pde4d	Zfp36l2
Gm19757	Per1	
Gna13	Pgm2l1	
	Phlda1	

Supplementary Fig. 3. iNKT cell lung signature genes. a. Heat map of normalized read counts from RNA-seq for genes with significantly elevated reads (raw p value of <0.1 calculated by DESeq2) in lung samples in all pairwise comparisons between lung and other tissue samples for each iNKT cell subset. Included are the mean normalized reads of NKT1 and NKT17 cells prepared from lungs of individual mice, designated with letters A-H, and from pooled mice (4-7 experimental replicates). Alphabetical list of lung signature genes (right).

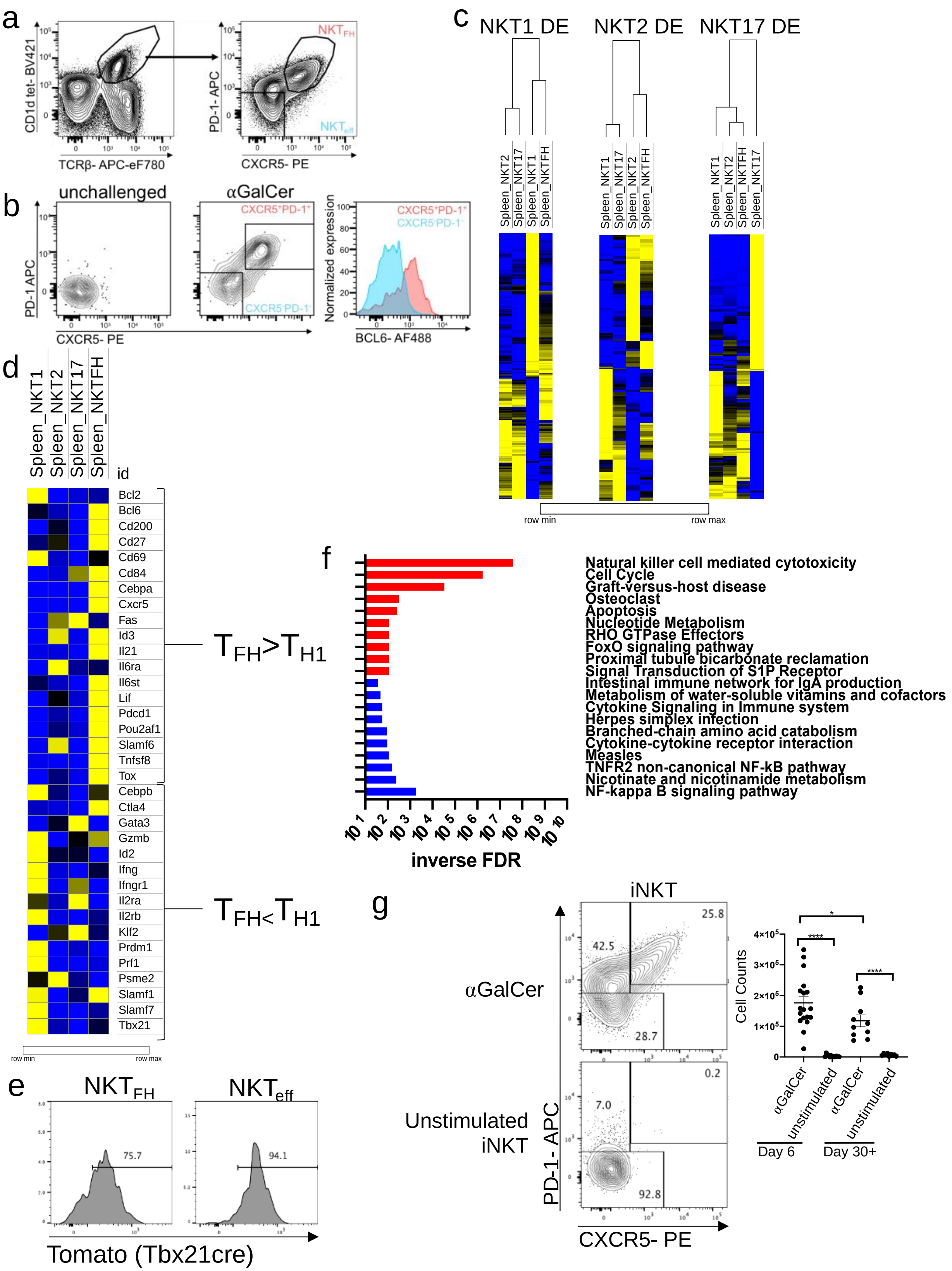

a
CD1d tet- BV421
TCRβ- APC-eF780
PD-1- APC
CXCR5- PE
NKTFH
NKTeff
b
unchallenged
αGalCer
PD-1 APC
CXCR5- PE
CXCR5+PD-1+
CXCR5-PD-1-
Normalized expression
BCL6- AF488
c
NKT1 DE
NKT2 DE
NKT17 DE
Spleen_NKT2
Spleen_NKT17
Spleen_NKT1
Spleen_NKTFH
row min
row max
d
Spleen_NKT1
Spleen_NKT2
Spleen_NKT17
Spleen_NKTFH
id
Bcl2
Bcl6
Cd200
Cd27
Cd69
Cd84
Cebpa
Cxcr5
Fas
Id3
Il21
Il6ra
Il6st
Lif
Pdcd1
Pou2af1
Slamf6
Tnfsf8
Tox
Cebpb
Ctla4
Gata3
Gzmb
Id2
Ifng
Ifngr1
Il2ra
Il2rb
Klf2
Prdm1
Prf1
Psme2
Slamf1
Slamf7
Tbx21
TFH>TH1
TFH<TH1
row min
row max
e
NKTFH
NKTeff
75.7
94.1
Tomato (Tbx21cre)
f
Natural killer cell mediated cytotoxicity
Cell Cycle
Graft-versus-host disease
Osteoclast
Apoptosis
Nucleotide Metabolism
RHO GTPase Effectors
FoxO signaling pathway
Proximal tubule bicarbonate reclamation
Signal Transduction of S1P Receptor
Intestinal immune network for IgA production
Metabolism of water-soluble vitamins and cofactors
Cytokine Signaling in Immune system
Herpes simplex infection
Branched-chain amino acid catabolism
Cytokine-cytokine receptor interaction
Measles
TNFR2 non-canonical NF-kB pathway
Nicotinate and nicotinamide metabolism
NF-kappa B signaling pathway
inverse FDR
g
iNKT
αGalCer
Unstimulated iNKT
25.8
42.5
28.7
0.2
7.0
92.8
PD-1- APC
CXCR5- PE
Cell Counts
αGalCer
unstimulated
αGalCer
unstimulated
Day 6
Day 30+

Supplementary Fig. 4. Phenotype and transcriptome of NKT_{FH} cells. a. Splenocytes from 7-week-old C57BL/6J females injected with αGalCer 6 days prior to analysis were enriched for iNKT cells and stained as described in Methods. Plots show the staining of live, lymphocyte-gated cells with anti-TCRβ and αGalCer loaded CD1d tetramers (left), and the staining of CD1d-tetramer$^+$ TCR β^+ cells for CXCR5 and PD-1 (right). b. Staining for expression of PD-1 and CXCR5 in gated iNKT cells from the spleen of unchallenged mice (left panel) and αGalCer injected mice (middle panel). Right panel: Staining for BCL6 in gated iNKT cells from immunized mice that were putative NKT_{FH}, or CXCR5$^+$ PD-1$^+$ (pink histogram) or that were double negative for these markers (NKT_{eff}, blue histogram). Data are representative of samples from 12 mice analyzed in three independent experiments. c. Heat map depicting the relative RNA-seq normalized read counts in splenic NKT1, NKT2, NKT17 and NKT_{FH} cells of genes that were DE in either NKT1 (left), NKT2 (center) or NKT17 cells (right) (Padj <0.1, shrunken lfc >1 or <-1). d. Heat map of RNA-seq normalized read counts from selected genes known to be DE in T_{FH} compared to T_{H1} cells. e. Expression of reporter in T-bet fate-mapping mice by NKT_{FH} and NKT_{eff} cells 3 days-post antigen exposure. f. ConsensusPathDB gene clustering: red up, blue down. g. NKT_{FH} persist in the spleen for at least one month. Left: representative flow cytometry showing NKT_{FH} (TCR β^+ CD1d-tetramer$^+$CXCR5$^+$PD-1$^+$) at d30+ after αGalCer plus unstimulated controls. Right: number of NKT_{FH} at day 6 or day 30+ after αGalCer plus controls. Data are combined from 14 experiments, n = 18 (d6 αGalCer), n = 13 (d6 unstimulated), n = 10 (d30 αGalCer), n = 9 (d30+ unstimulated), error bars depict SEM. Mann-Whitney test (two-tailed), **** = p values of <0.0001, * = p value of 0.04.

Supplementary References

1. Engel, I. *et al.* Innate-like functions of natural killer T cell subsets result from highly divergent gene programs. *Nature Immunology* **17**, 728-739 (2016).
