# Supplementary material for: Transcriptome and chromatin landscape of iNKT cells are shaped by subset differentiation and antigen exposure
Source: Nat Commun. 2021 Mar 4;12:1446. doi: 10.1038/s41467-021-21574-w (PMC7933435; doi:10.1038/s41467-021-21574-w)
Supplement: Supplementary file 3 — Reporting Summary [file 41467_2021_21574_MOESM3_ESM.pdf]

nature research

Corresponding author(s): James P. Scott-Browne, Mitchell Kronenberg

Last updated by author(s): Dec 8, 2020

Reporting Summary

Nature Research wishes to improve the reproducibility of the work that we publish. This form provides structure for consistency and transparency in reporting. For further information on Nature Research policies, see our Editorial Policies and the Editorial Policy Checklist.

Statistics

For all statistical analyses, confirm that the following items are present in the figure legend, table legend, main text, or Methods section.

n/a	Confirmed	
☐	☒	The exact sample size (*n*) for each experimental group/condition, given as a discrete number and unit of measurement
☐	☒	A statement on whether measurements were taken from distinct samples or whether the same sample was measured repeatedly
☐	☒	The statistical test(s) used AND whether they are one- or two-sided<br>*Only common tests should be described solely by name; describe more complex techniques in the Methods section.*
☒	☐	A description of all covariates tested
☐	☒	A description of any assumptions or corrections, such as tests of normality and adjustment for multiple comparisons
☐	☒	A full description of the statistical parameters including central tendency (e.g. means) or other basic estimates (e.g. regression coefficient) AND variation (e.g. standard deviation) or associated estimates of uncertainty (e.g. confidence intervals)
☒	☐	For null hypothesis testing, the test statistic (e.g. *F*, *t*, *r*) with confidence intervals, effect sizes, degrees of freedom and *P* value noted<br>*Give P values as exact values whenever suitable.*
☒	☐	For Bayesian analysis, information on the choice of priors and Markov chain Monte Carlo settings
☒	☐	For hierarchical and complex designs, identification of the appropriate level for tests and full reporting of outcomes
☒	☐	Estimates of effect sizes (e.g. Cohen's *d*, Pearson's *r*), indicating how they were calculated

Our web collection on statistics for biologists contains articles on many of the points above.

Software and code

Policy information about availability of computer code

Data collection	Flow cytometry data was collected with BD FACSDIVA
Data analysis	Flow cytometry data was analyzed with FlowJo Software (10.6.1, FlowJo LLC, BD Life Sciences) and Prism 7 (GraphPad Software)<br><br>RNA-seq (complete description provided in Methods)<br>TopHat (v 1.4.1), PRINSEQ Lite (v 0.20.3) SAMtools (Li, H. et al. The Sequence Alignment/Map format and SAMtools. Bioinformatics 25, 2078-2079 (2009)), htseq-count program (v 0.7.1), R/Bioconductor package DESeq2 (v 1.6.3), R/Bioconductor package EdgeR, Principal Component Analysis (PCA) was performed using the 'prcomp' function in R. Data were also analyzed using the Pre-ranked Gene Set Enrichment Analysis algorithm (Broad Institute and University of California) and the Consensus Path Database platform (Max Planck Institute).<br><br>ATAC-seq (complete description provided in Methods)<br>Bowtie (v 1.0.0), tim_galore (v 0.3.8), picard MarkDuplicates (1.94), MEDIPS (v1.24.0, Java Genomics Toolkit (1.1.0), MACS2 (v2.1.1.20160309), chromVAR.

For manuscripts utilizing custom algorithms or software that are central to the research but not yet described in published literature, software must be made available to editors and reviewers. We strongly encourage code deposition in a community repository (e.g. GitHub). See the Nature Research guidelines for submitting code & software for further information.

Data

Policy information about availability of data

All manuscripts must include a data availability statement. This statement should provide the following information, where applicable:

- Accession codes, unique identifiers, or web links for publicly available datasets
- A list of figures that have associated raw data
- A description of any restrictions on data availability

The sequences used in this article have been submitted to the Gene Expression Omnibus with accession number (GSE161492) https://www.ncbi.nlm.nih.gov/geo/query/acc.cgi?acc=GSE161492.
ATAC-seq sequence data associated figures: Figure 1; Figure 2a, b, c; Figure 3 a, c; Figure 4a; Figure 5 a, b, c; Figure 6a.
RNA-seq sequence data associated figures: Figure 2d; Figure 3b; Figure 4 b, c, d, e; Figure 5 d, e, f; Supplementary Figure 1d; Supplementary Figure 2; Supplementary Figure 3; Supplementary Figure 4 c, d,f.

There are no restrictions on data availability. Additional information and materials will be made available upon request.

Field-specific reporting

Please select the one below that is the best fit for your research. If you are not sure, read the appropriate sections before making your selection.

- [x] Life sciences
- [ ] Behavioural & social sciences
- [ ] Ecological, evolutionary & environmental sciences

For a reference copy of the document with all sections, see nature.com/documents/nr-reporting-summary-flat.pdf

Life sciences study design

All studies must disclose on these points even when the disclosure is negative.

Sample size	No sample size calculations were performed. Sample size was determined to be adequate based on the magnitude and consistency of measurable differences between groups, as well as the feasibility of performing highly technical experiments with a rare cell population.
Data exclusions	Post-sequencing, stringent, pre-established quality controls were applied and samples that failed quality control standards were eliminated from further analysis.
Replication	Replicate samples were taken from at least two experiments for all experiments described; similar results were seen in samples sequenced from different experiments. For flow cytometric experiments, data were successfully repeated and were drawn from analysis of multiple animals.
Randomization	Not relevant for this study. Mice were gender- and age-matched.
Blinding	The investigators were not blinded during data collection. However, RNA-seq data and ATAC-seq samples were processed by separate scientists and each data set was analyzed by separate bioinformaticians.

Reporting for specific materials, systems and methods

We require information from authors about some types of materials, experimental systems and methods used in many studies. Here, indicate whether each material, system or method listed is relevant to your study. If you are not sure if a list item applies to your research, read the appropriate section before selecting a response.

Materials & experimental systems

n/a	Involved in the study	
[ ]	[x]	Antibodies
[x]	[ ]	Eukaryotic cell lines
[x]	[ ]	Palaeontology and archaeology
[ ]	[x]	Animals and other organisms
[x]	[ ]	Human research participants
[x]	[ ]	Clinical data
[x]	[ ]	Dual use research of concern

Methods

n/a	Involved in the study	
[x]	[ ]	ChIP-seq
[ ]	[x]	Flow cytometry
[x]	[ ]	MRI-based neuroimaging

Antibodies

Antibodies used	For depletion studies, the following biotinylated antibodies were used: CD8a (53–6.7, Tonbo Biosciences, lot C0081073117304), CD19 (1D3, Tonbo Biosciences, lot C0193082217304), CD24 (M1/69, BD Biosciences, lot 8290955), CD62L (MEL-14, Invitrogen, lot E034527), CD11b (M1/70, Tonbo Biosciences, lot C0112020218304), CD11c (N418, Tonbo Biosciences, lot C0114032117304), F4/80 (BM5.1, Tonbo Biosciences, lot C4801073117304), EpCam (G8.8, Biolegend, lot BB273843), and TER-119 (TER-119, Tonbo Biosciences, lot C592106417304).<br>For sorting and flow cytometry:<br>anti-TCRb-APC-eF780 (H57-597, ThermoFisher Scientific, lot 2114197), anti-CD8a-PE CF594 (53-6.7, BD Biosciences, lot 9044592), anti-CD19-PE CF594 (1D3, BD Biosciences, lot 3151636), anti-CD4-AF700 (GK1.5, BioLegend, lot B240053), anti-IL-17RB-AF488 (FAB10402G, R&D Systems, lot ACWT0115101), anti-ICOS-PerCP Cy5.5 (C398.4A, BioLegend, lot B237032), anti-CD122-BV650 (5H4, BD Biosciences, lot 8313944), anti-CXCR3-APC (CXCR3-173, BioLegend, lot B233224), anti-SDC1-PE (281-2, BioLegend, lot BB203320), anti-FR4-PE Cy7 (ebio12A5, ThermoFisher Scientific, lot 4302658), anti-CXCR5-PE (clone L138D7, BioLegend, lot B168296), anti-PD-1-APC (clone RMP1-30, BioLegend, lot B145236), anti-TCRgd-FITC (clone GL3, BD Biosciences, lot 8290551), anti-NK1.1-PE Cy7 (clone PK136, BD Biosciences, lot 552878), anti-PLZF-AF647 (clone R17–809, lot 9070702, BD Biosciences), anti-T-bet-AF488 (clone O4–46, lot 9093594, BD Biosciences), anti-RORgt-PE-CF594 (clone Q31-378, lot 9073662, BD Biosciences), anti-BCL-6-AF488 (clone K112-91, lot 8012766, BD Biosciences), anti-KLRG1- PECy7 (clone 2F1, lot 1982690, Thermo-Fisher), anti-CTLA-4-PE (clone UC10-4B9, lot E01527-196, Thermo-Fisher), anti-CX3CR1-BV711 (clone SA011F11, BioLegend, lot B202222).
Validation	Validation statements are provided on the manufacturers' websites and we have used them in many studies over many years.

Animals and other organisms

Policy information about studies involving animals; ARRIVE guidelines recommended for reporting animal research

Laboratory animals	C57BL/6J female or male mice aged 6-8 weeks old were were used for all experiments, with the exception of Tbx21creRosa26-loxPSTOP-<br>loxP-tdTomato mice female and male mice aged 6-8 weeks.
Wild animals	No wild animals were used in these studies
Field-collected samples	No field samples were collected
Ethics oversight	All studies were approved by the Institutional Animal Care and Use Committee at the La Jolla Institute for Immunology.

Note that full information on the approval of the study protocol must also be provided in the manuscript.

Flow Cytometry

Plots

Confirm that:

- [x] The axis labels state the marker and fluorochrome used (e.g. CD4-FITC).
- [x] The axis scales are clearly visible. Include numbers along axes only for bottom left plot of group (a 'group' is an analysis of identical markers).
- [x] All plots are contour plots with outliers or pseudocolor plots.
- [x] A numerical value for number of cells or percentage (with statistics) is provided.

Methodology

Sample preparation	Following euthanasia, thymus tissue and spleens were removed, lungs and livers were perfused with 3-10 mL of liver perfusion medium (Gibco) until tissues cleared. Livers were mashed through a 70 uM nylon filter (Fisher). Liver lymphocytes were isolated by centrifugation at 850xg in 37.5% Percoll for 20 minutes. Lungs were placed in GentleMacs C tubes (Miltenyi Biotec) with 2 mL Spleen Dissociation Medium (STEMCELL Technologies) and homogenized using the Miltenyi GentleMacs dissociator. Following homogenization, suspensions were filtered with a 70 uM filter and washed twice with RPMI + 10% FBS. Thymus and spleens were homogenized through a 70 uM nylon filter and washed with RPMI + 10% FBS.
Instrument	FACSAria III, FACSAria Fusion, Fortessa analyzer (BD Biosciences)
Software	Flow cytometry data collected with BD FACSDIVA software. Flow cytometry data analyzed with FlowJo 10.6.1 (FlowJo LLC, BD Life Sciences) and Prism 7 (GraphPad Software)
Cell population abundance	For RNA-seq, 400 cell aliquots were collected. For ATAC-seq, 10,000 cell aliquots were collected. For flow cytometric analyses, 6x10^5 events were collected for lung samples, otherwise 1 to 2 million total events were collected for analysis.
Gating strategy	For sorting and flow cytometric analysis of NKT subsets from thymus and peripheral tissues, cells were sorted based on the following strategy: sorting lymphocytes (by FSC/SSC): Singlets (FSC-H/FSC-W: SSC-H/SSC-W): live (viability dye low)/CD19- and CD8-: tetramer+/TCRb+. Subsets were defined as follows (gating strategy provided in supplementary figure 1): NKT1: CXCR3 +ICOS-CD122+SDC1-IL-17RB-; NKT2: CXCR3-ICOS+CD122-SDC1-IL-17RB+CD4+FR4+; NKT17: CXCR3-ICOS+CD122-SDC1+For

sorting of gd T cells, CD4+ T cells, NK cells, and iNKT cells from lung and spleen, populations were sorted based on the following gating strategy (post iNKT-gating shown in Supplementary Figure 4) sorting lymphocytes (by FSC/SSC), singlets (FSC-H/FSC-W and SSC-H/SSC-W): live (viability dye negative): CD8- and CD19-, and from this point sorting iNKT cells as CD1d-tetramer-binding, TCRb+; gd T cells as TCRb-TCRgd+, NK cells as TCRb-, NK1.1+, TCRgd- and CD4+ T cells as TCRb+CD4+.

☒ Tick this box to confirm that a figure exemplifying the gating strategy is provided in the Supplementary Information.
